# Long-Term Effects of Maternal Deprivation on the Neuronal Soma Area in the Rat Neocortex

**DOI:** 10.1155/2014/235238

**Published:** 2014-05-08

**Authors:** Milan Aksić, Nevena V. Radonjić, Dubravka Aleksić, Gordana Jevtić, Branka Marković, Nataša Petronijević, Vidosava Radonjić, Branislav Filipović

**Affiliations:** ^1^Institute of Anatomy “Niko Miljanic”, Faculty of Medicine, University of Belgrade, dr Subotića 4, Belgrade 11000, Serbia; ^2^Institute of Clinical and Medical Biochemistry, Faculty of Medicine, University of Belgrade, Pasterova 2, Belgrade 11000, Serbia; ^3^Faculty of Sport and Physical Education, University of Belgrade, Blagoja Parovića 156, Belgrade 11000, Serbia

## Abstract

Early separation of rat pups from their mothers (*separatio a matrem*) is considered and accepted as an animal model of perinatal stress. Adult rats, separated early postnatally from their mothers, are developing long-lasting changes in the brain and neuroendocrine system, corresponding to the findings observed in schizophrenia and affective disorders. With the aim to investigate the morphological changes in this animal model we exposed 9-day-old (P9) Wistar rats to a 24 h maternal deprivation (MD). At young adult age rats were sacrificed for morphometric analysis and their brains were compared with the control group bred under the same conditions, but without MD. Rats exposed to MD had a 28% smaller cell soma area in the prefrontal cortex (PFCX), 30% in retrosplenial cortex (RSCX), and 15% in motor cortex (MCX) compared to the controls. No difference was observed in the expression of glial fibrillary acidic protein in the neocortex of MD rats compared to the control group. The results of this study demonstrate that stress in early life has a long-term effect on neuronal soma size in cingulate and retrosplenial cortex and is potentially interesting as these structures play an important role in cognition.

## 1. Introduction

Schizophrenia is a severe psychiatric disorder affecting 0.5–1% of general population. It is clinically characterised by disturbed thought processes, delusions, hallucinations, and reduced social skills [1]. The neuropathological and neuroanatomical findings in patients with schizophrenia have been proposed to arise from dysfunction of structural reorganization during early brain development [2, 3] or postnatally from altered maturation of synaptic elimination [4]. In the patients with first episode of schizophrenia several morphological observations have been described: reduced cortical thickness of anterior cingulate [5] and prefrontal cortex [6], enlarged ventricles, decrease in size of ventromedial temporal lobe structures, parahippocampal cortical thickness, and increase in the gyrification index [7–15]. Morphometric microscopy studies revealed alterations in neuronal density, size and shape in limbic, and temporal and frontal cortical regions [7, 16–25]. Moreover, there is growing evidence that patients with chronic schizophrenia have reduced cortical thickness predominantly in frontotemporal regions [26, 27].

The plethora of clinical evidence implies that schizophrenia has a neurodevelopmental component [28, 29] and has led to the development of novel animal models, focusing specifically on the long-term consequences of early environmental manipulations. Single 24-hour period of maternal deprivation (MD) of rat pups at postnatal day 9 (P9) leads to disturbances in prepulse inhibition and latent inhibition [30, 31] and induces neurochemical changes in brain structures implicated in the neuropathology of schizophrenia [32, 33].

The aim of this study was to elucidate long-term effects of maternal deprivation on the rat brain morphology. We have studied the areas important for information processing such as motor, prefrontal, and retrosplenial cortex. We observed reduced cell soma area of neurons in the cortex of MD animals compared to the controls. However, no difference was observed in expression of GFAP astrocyte marker. These results suggest that early life stress can alter brain morphology and consequently impact its function.

## 2. Materials and Methods

### 2.1. Animals and Procedures

Male and four nulliparous female Wistar rats 3-month-old were put together in standard plexiglass cages with sawdust (26 × 42 × 15 cm), in a temperature controlled room (23 ± 1°C). The rats were on a standard 12 h light/dark cycle with lights on from 7:00 to 19:00 h, with water and food available* ad libitum*. Two weeks later, males were removed and the dams were checked twice daily for delivery. The day of delivery was denoted as a P0. On P9, two litters were subjected to the maternal deprivation procedure according to the previously published protocol [31, 34]. Briefly, dams were removed from the litter at 10:00 am, after which the pups were weighed and returned to their home cage. They remained in their home cage at room temperature for 24 h. On P10, the pups were weighed again and dams were returned to their cages. The dams of the control litters were briefly (3 min) removed from their home cages and the pups were weighed on both P9 and P10. All litters were later left undisturbed except for the routine cleaning of the cages until P21 when litters were weaned and classified according to sex. For morphological and biochemical studies only male rats were used in order to avoid sexual dimorphism [35] and many of the previous studies were performed on males [36, 37]. Animals were sacrificed at the period of young adulthood (P60). All efforts were made to minimize animal suffering and reduce the number of animals used in the study. All experiments were carried out according to the NIH Guide for Care and Use of Laboratory Animals and were approved by the Local Bioethics Committee.

### 2.2. Tissue Processing and Immunohistochemistry Staining

For morphological analysis, five male animals from the control and experimental group were anaesthetized with chloral hydrate (3 mg/kg, i.p.) and transcardially perfused with the fixative (4% formaldehyde in 0.1 M phosphate buffer). The brains were postfixed for 24 h at +4°C and cryoprotected by infiltration with sucrose for 2 days at 4°C (20% sucrose in 0.1 M phosphate buffer). Brains were frozen by immersion in 2-methyl-butane (Sigma-Aldrich, St. Louis, MO) precooled to −80°C and stored at −80°C until cutting. Serial transverse sections (25 *μ*m thick) were cut on a cryostat (Leica Instruments, Nußloch, Germany). Sections were collected on SuperFrost Plus glass slides (Menzel, Braunschweig, Germany) in a standard sequence so that four sections 250 *μ*m apart were present on each slide. Immunohistochemical staining was performed after water-bath antigen retrieval in 0.01 M sodium citrate solution, pH 9.0, for 30 min at 80°C. Nonspecific binding was blocked using 5% normal goat serum, dissolved in phosphate buffered saline (PBS), pH 7.3, and supplemented with 0.2% Triton X-100, 0.02% sodium azide for 1 h at RT. Incubation with the primary NeuN antibody (mouse monoclonal NeuN antibody, 1 : 1000; Millipore, Schwalbach, Germany), diluted in PBS containing 0.5% lambda-carrageenan (Sigma-Aldrich) and 0.02% sodium azide, was carried out for 2 days at 4°C. After washing in PBS (3 × 15 min at RT), the endogenous peroxidase activity was blocked by submerging sections in 3% H_2_O_2_ solution for 10 min. The sections were then incubated for 30 min at RT with EnVision + Dual Link System-HRP (Dako, Carpinteria, CA). After a subsequent wash in PBS, the sections were incubated with diaminobenzidine with chromogen (Dako, Carpinteria, CA) for approximately 20 min, until the immune reaction was visible. Finally, the sections were counterstained in Mayer's hematoxylin (Fisher Scientific, Leicestershire, UK) for 30 sec, rinsed with PBS, dehydrated, and mounted with DPX (Sigma Aldrich). Specificity of staining was controlled by replacing the primary antibody with the normal serum from the animal in which the antibody was produced, which resulted in the absence of signal.

### 2.3. Estimations of Cells Soma Area of NeuN Immunolabeled Neurons

Estimations of NeuN-positive (NeuN+) cells soma area were performed at the level of the largest cell body cross-sectional area. Coronal brain sections stained for NeuN were selected for analyses. Four sections 250 *μ*m apart were analyzed per animal. NeuN immunolabeled neurons were identified by their position in the prefrontal, retrosplenial, and motor cortex. The sample size was between 20 and 30 neurons per animal. Areas were measured using the ImageTool 2.0 (University of Texas, San Antonio, TX).

### 2.4. Image Acquisition and Quantitative Analysis of Immunolabeled Neurons

Pictures were taken on optical microscope (DM4000 Leica) with a 40x objective and analyzed in Photoshop 7.0 software (Adobe, San Jose, CA), using a 1 cm grid. NeuN-immunoreactive cells were counted in stereological sections of the rat brains on the same distance from bregma (2.52 mm for prefrontal cortex and −2.76 mm for retrosplenial and motor cortex). The counted number of NeuN-immunoreactive cells was expressed per unit area (*μ*m^2^), and we will further refer to it as a profile density. At least 200 random microscope fields (area 20 *μ*m^2^) were counted in the retrosplenial, motor cortex and prefrontal cortex of each section.

### 2.5. Quantitative Western Blot Analysis

For Western blot analysis, five male animals from the control and experimental groups (P60) were killed by cervical dislocation and decapitation without anesthesia. After decapitation, the brains were quickly removed and transferred to liquid nitrogen. The dorsolateral frontal cortex (4.2 mm up to −1.32 mm from bregma; [38]) was homogenized in lysis buffer (50 mM Tris-HCl pH 7.4, 150 mM NaCl, 1% NP-40, 1 mM phenylmethylsulfonyl fluoride, and protease inhibitor cocktail) on ice for 30 min, centrifuged at 18.000 g for 15 min at 4°C, and the supernatant was collected. An equal amount of protein from each sample was separated by sodium dodecyl sulfate-polyacrylamide gel electrophoresis (SDS-PAGE) on a 10% gel and transferred to a nitrocellulose membrane (Amersham, Buckinghamshire, UK). Rabbit polyclonal anti-GFAP primary antibody (Dako, Denmark) was used. All membranes were stripped and reprobed with anti-actin antibody (Sigma-Aldrich) to ensure equal loading. Western blots were scanned and densitometric analysis was performed using ImageQuant 5.2 (GE Healthcare, Buckinghamshire, UK).

### 2.6. Statistical Analysis

All numerical data are presented as group mean values with standard errors of the mean (SEM). Morphological analysis was performed bilaterally, and if no difference was observed data were pooled together for presentation of results. All comparisons were performed by the Student's *t*-test, and the threshold value for acceptance of the difference was 5%.

## 3. Results

### 3.1. Cell Soma Areas of NeuN Immunolabeled Neurons in Prefrontal, Retrosplenial, and Motor Cortex

**Figure 1:**
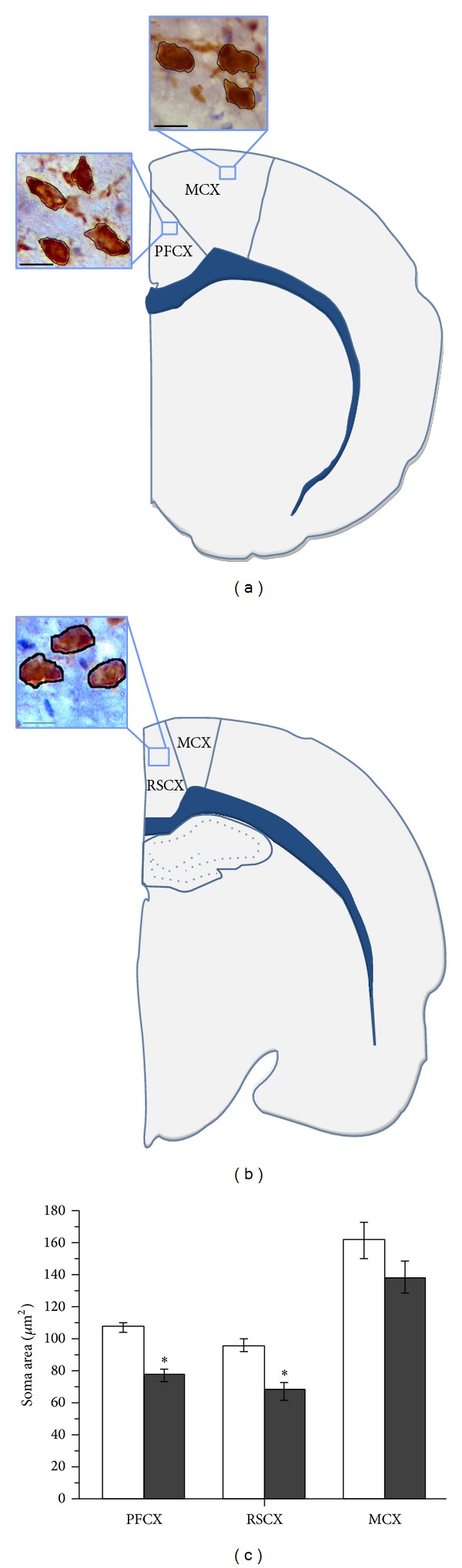
Cell soma area of NeuN-positive cells. Schematic representation of prefrontal (cingulate) cortex (PFCX) (a), motor cortex (MCX) (a, b), and retrosplenial cortex (RSCX) (b) on coronal sections of adult rat brain (according to Paxinos and Watson, 2005). Insets: NeuN cells on high magnification; scale bar 5 *μ*m. (c) Profile densities of NeuN-immunolabeled cells in the PFCX, RSCX, and MCX of MD and control rats. Results are presented as the mean values ± SEM.

Previously, we have demonstrated that MD long-term results in decreased cortical thickness [39]. Here, we explored if this reduction is due to a decrease in size of neuronal soma area. We measured the cell soma area of the NeuN-positive (NeuN+) cells in retrosplenial, prefrontal, and motor cortex. The cell soma area of the NeuN+ cells in the control group of rats was 107.9 ± 2.8 *μ*m^2^ in prefrontal cortex, 97 ± 4.2 *μ*m^2^ in retrosplenial cortex, and 163.7 ± 10.4 *μ*m^2^ in motor cortex (Figure 1). In the MD group, the cell soma area of NeuN+ cells was 78.1 ± 5.1 *μ*m^2^ in prefrontal, 68.5 ± 6.1 *μ*m^2^ in retrosplenial, and 139.4 ± 9.8 *μ*m^2^ in motor cortex (Figure 1). Analysis of the obtained results by *t*-test showed that this difference is statistically significant in prefrontal and retrosplenial cortex demonstrating stressful effect of maternal separation (PFCX (*P* = 0.002), RSCX (*P* = 0.005), MCX (*P* = 0.1)) (Figure 1).

### 3.2. Expression of GFAP Protein in Neocortex

**Figure 2:**
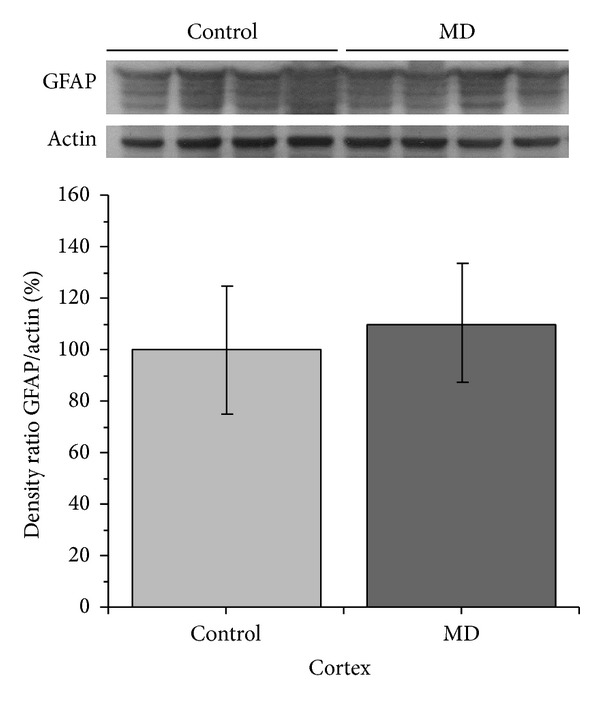
Expression of GFAP in neocortex homogenates. Figure is accompanied by representative immunoblots from the same gel. Results are presented as the mean values ± SEM.

Next, we examined the expression of astrocyte marker, GFAP, in neocortex of MD animals and control group (Figure 2). Quantitative analysis of the immunoblot data did not show difference in GFAP expression in the neocortex (*P* > 0.05) (Figure 2). We can conclude that in neocortex substantial loss of neurons occurs in animals stressed by maternal deprivation and the levels of GFAP expression in astrocytes are not affected.

## 4. Discussion

In this study we demonstrate that MD used as a perinatal stressor has long-term effects on neuronal cell soma area and does not affect expression of astrocyte marker in the rat neocortex. Stress is an unavoidable part of human existence. Extreme forms of acute and chronic stress may cause an abnormal mental state and affect behaviour and represent risk factors for psychiatric disorders such as schizophrenia and mood disorders [40, 41]. Previously, we have demonstrated in maternally deprived animals reduced thickness of retrosplenial, prefrontal, and motor cortex and decreased density of neurons in retrosplenial and prefrontal cortex [39]. Also, we observed reduced expression of neuronal marker NeuN in the neocortex of MD rats [39]. Here, we further analyze this phenomenon and observe reduction in the neuronal cell soma area in the prefrontal and retrosplenial cortex. No difference in the neuronal soma size was observed in motor cortex.

Cerebral cortex and hippocampus play a central role in cognition and memory. Hyde and Crook [42] proposed that focal pathological changes in either the prefrontal cortex or mesial temporal lobe could lead to neurochemical changes in multiple neurotransmitter systems such as dopaminergic, glutamatergic, and cholinergic. Recently, our group demonstrated region-specific changes in the activity of acetylcholine esterase (AChE) and density of cholinergic fibers as a result of perinatal maternal deprivation [43].

The prefrontal cortex has been described as a region susceptible to detrimental effects of the exposure to chronic stress [44] and the most promising brain region in the terms of prediction of later psychosis [45]. The most consistent cognitive findings are those testing prefrontal cortical function, such as spatial working memory, antisaccade eye movements, olfactory identification, and tasks requiring rapid processing of information such as story recall [45]. Functional imaging studies showed structural and functional impairments in PFCX of patients with psychiatric disorders, especially in patients exposed to childhood maltreatment [46] or harsh corporal punishment [47]. Postmortem brain studies of schizophrenia patients demonstrated smaller neuron size and neuronal density in the cingulate cortex [22, 23]. Previous studies of PFCX in maternally deprived rats observed numerous impairments in neuronal activity [48, 49], dendritic morphology [50–52], and protein expression [53, 54].

Growing evidence indicates that entorhinal cortex, which has an important role in declarative memory, might be affected in patients with schizophrenia. Postmortem brain studies of schizophrenia patients revealed differences in neuron density, size, and arrangement, abnormalities in synapse-related proteins, alterations in monoaminergic and glutamatergic innervation, and receptor distribution and abnormalities in the expression of cytoskeletal proteins [55].

Lately, epigenetic factors are in the focus of etiological studies of psychiatric disorders. Cues from the social and physical environments in early life are considered to induce variations in epigenetic programing that functions as an adaptive response of the genome to the anticipated environment [56]. In mammalian development, the perinatal period represents a critical period when epigenetic programs are laid down resulting in changes in gene expression and to long-term influences on brain development and behavior. Recently, studies of non-human primate,* Rhesus Macaque*, revealed association of early maternal deprivation with DNA hydroxymethylation changes of promoters of genes in the adult monkey cortex related to neurological functions and psychological disorders [57]. In this study, we have characterised morphological changes induced by maternal deprivation in adult rat cortex. Further studies are necessary to clarify if MD used in this animal model results in epigenetic modulations.

## 5. Conclusion

In conclusion, this is the first study to provide evidence that early stress caused by MD in rats leads to alteration in size of neuronal cell soma area in the retrosplenial and prefrontal cortex and does not affect expression of astrocyte marker GFAP in the neocortex. These results further contribute to characterisation of MD model of animal perinatal stress and are potentially interesting as these structures play an important role in cognition.

## References

[1] Andreasen NC (1995). Symptoms, signs, and diagnosis of schizophrenia. *The Lancet*.

[2] Waddington JL (1993). Schizophrenia: developmental neuroscience and pathobiology. *The Lancet*.

[3] Weinberger DR (1995). From neuropathology to neurodevelopment. *The Lancet*.

[4] Feinberg I (1982). Schizophrenia: caused by a fault in programmed synaptic elimination during adolescence?. *Journal of Psychiatric Research*.

[5] Fornito A, Yücel M, Wood SJ (2008). Surface-based morphometry of the anterior cingulate cortex in first episode schizophrenia. *Human Brain Mapping*.

[6] Venkatasubramanian G, Jayakumar PN, Gangadhar BN, Keshavan MS (2008). Automated MRI parcellation study of regional volume and thickness of prefrontal cortex (PFC) in antipsychotic-naïve schizophrenia. *Acta Psychiatrica Scandinavica*.

[7] Arnold SE, Trojanowski JQ (1996). Recent advances in defining the neuropathology of schizophrenia. *Acta Neuropathologica*.

[8] Bogerts B, Falkai P, Greve B, Heckers S, Heinsen H, Beckmann H (1991). Evidence of reduced temporolimbic structure volumes in schizophrenia. *Archives of General Psychiatry*.

[9] Buchsbaum MS (1990). Frontal lobes, basal ganglia, temporal lobes—three sites for schizophrenia?. *Schizophrenia Bulletin*.

[10] Degreef G, Bogerts B, Falkai P (1992). Increased prevalence of the cavum septum pellucidum in magnetic resonance scans and post-mortem brains of schizophrenic patients. *Psychiatry Research—Neuroimaging*.

[11] DeLisi LE, Dauphinais ID, Gershon ES (1988). Perinatal complications and reduced size of brain limbic structures in familial schizophrenia. *Schizophrenia Bulletin*.

[12] Frith CD (1997). Functional brain imaging and the neuropathology of schizophrenia. *Schizophrenia Bulletin*.

[13] Harvey I, Ron MA, Du Boulay LG, Wicks D, Lewis SW, Murray RM (1993). Reduction of cortical volume in schizophrenia on magnetic resonance imaging. *Psychological Medicine*.

[14] Honer WG, Bassett AS, Falkai P, Beach TG, Lapointe JS (1996). A case study of temporal lobe development in familial schizophrenia. *Psychological Medicine*.

[15] Kleinschmidt A, Falkai P, Huang Y, Schneider T, Furst G, Steinmetz H (1994). In vivo morphometry of planum temporale asymmetry in first-episode schizophrenia. *Schizophrenia Research*.

[16] Falkai P, Bogerts B (1986). Cell loss in the hippocampus of schizophrenics. *European Archives of Psychiatry and Neurological Sciences*.

[17] Kovelman JA, Scheibel AB (1984). A neurohistological correlate of schizophrenia. *Biological Psychiatry*.

[18] Scheibel AB, Kovelman JA (1981). Disorientation of the hippocampal pyramidal cell and its processes in the schizophrenic patients. *Biological Psychiatry*.

[19] Zaidel DW, Esiri MM, Harrison PJ (1997). The hippocampus in schizophrenia: lateralized increase in neuronal density and altered cytoarchitectural asymmetry. *Psychological Medicine*.

[20] Akbarian S, Bunney WE, Potkin SG (1993). Altered distribution of nicotinamide-adenine dinucleotide phosphate-diaphorase cells in frontal lobe of schizophrenics implies disturbances of cortical development. *Archives of General Psychiatry*.

[21] Akbarian S, Vinuela A, Kim JJ, Potkin SG, Bunney WE, Jones EG (1993). Distorted distribution of nicotinamide-adenine dinucleotide phosphate-diaphorase neurons in temporal lobe of schizophrenics implies anomalous cortical development. *Archives of General Psychiatry*.

[22] Benes FM, Bird ED (1987). An analysis of the arrangement of neurons in the cingulate cortex of schizophrenic patients. *Archives of General Psychiatry*.

[23] Benes FM, Majocha R, Bird ED, Marotta CA (1987). Increased vertical axon numbers in cingulate cortex of schizophrenics. *Archives of General Psychiatry*.

[24] Benes FM, McSparren J, Bird ED, SanGiovanni JP, Vincent SL (1991). Deficits in small interneurons in prefrontal and cingulate cortices of schizophrenic and schizoaffective patients. *Archives of General Psychiatry*.

[25] Qoldman-Rakic PS, Selemon LD (1997). Functional and anatomical aspects of prefrontal pathology in schizophrenia. *Schizophrenia Bulletin*.

[26] Kuperberg GR, Broome MR, McGuire PK (2003). Regionally localized thinning of the cerebral cortex in schizophrenia. *Archives of General Psychiatry*.

[27] Nesvåg R, Lawyer G, Varnäs K (2008). Regional thinning of the cerebral cortex in schizophrenia: effects of diagnosis, age and antipsychotic medication. *Schizophrenia Research*.

[28] Weinberger DR (1996). On the plausibility of “The neurodevelopmental hypothesis” of schizophrenia. *Neuropsychopharmacology*.

[29] Pilowsky LS, Kerwin RW, Murray RM (1993). Schizophrenia: a neurodevelopmental perspective. *Neuropsychopharmacology*.

[30] Ellenbroek BA, Cools AR (2002). Early maternal deprivation and prepulse inhibition: the role of the postdeprivation environment. *Pharmacology Biochemistry and Behavior*.

[31] Ellenbroek BA, Cools AR (1995). Maternal separation reduces latent inhibition in the conditioned taste aversion paradigm. *Neuroscience Research Communications*.

[32] Harrison PJ (1999). The neuropathology of schizophrenia. A critical review of the data and their interpretation. *Brain*.

[33] Weinberger DR (1999). Cell biology of the hippocampal formation in schizophrenia. *Biological Psychiatry*.

[34] Roceri M, Hendriks W, Racagni G, Ellenbroek BA, Riva MA (2002). Early maternal deprivation reduces the expression of BDNF and NMDA receptor subunits in rat hippocampus. *Molecular Psychiatry*.

[35] Woolley CS, McEwen BS (1992). Estradiol mediates fluctuations in hippocampal synapses density during the estrous cycle in the adult rat. *The Journal of Neuroscience*.

[36] Vivinetto AL, Suarez MM, Rivarola MA (2013). Neurogiological effects of neonatal maternal separation and post-weaning enviromental enrichment. *Behavioural Brain Research*.

[37] Own LS, Potel PD (2013). Maternal behavior and offspring resiliency to maternal separation in C57B/6 mice. *Hormones and Behavior*.

[38] Paxinos G, Watson C (2005). *The Rat Brain in Stereotaxic Coordinates*.

[39] Aksić M, Radonjić NV, Aleksić D (2013). Long-term effects of the maternal deprivation on the volume and number of neurons in the rat neocortex and hippocampus. *Acta Neurobiologiae Experimentalis*.

[40] Fone KCF, Porkess MV (2008). Behavioural and neurochemical effects of post-weaning social isolation in rodents-relevance to developmental neuropsychiatric disorders. *Neuroscience and Biobehavioral Reviews*.

[41] Leonard BE (2001). Stress, norepinephrine and depression. *Journal of Psychiatry and Neuroscience*.

[42] Hyde TM, Crook JM (2001). Cholinergic systems and schizophrenia: primary pathology or epiphenomena?. *Journal of Chemical Neuroanatomy*.

[43] Marković B, Radonjić NV, Aksić M, Filipović B, Petronijević N (2014). Long-term effects of maternal deprivation on cholinergic system in rat brain. *Biomed Research International*.

[44] Arnsten AFT (2009). Stress signalling pathways that impair prefrontal cortex structure and function. *Nature Reviews Neuroscience*.

[45] Wood SJ, Pantelis C, Velakoulis D, Yücel M, Fornito A, McGorry PD (2008). Progressive changes in the development toward schizophrenia: studies in subjects at increased symptomatic risk. *Schizophrenia Bulletin*.

[46] van Harmelen A, van Tol M, van der Wee NJA (2010). Reduced medial prefrontal cortex volume in adults reporting childhood emotional maltreatment. *Biological Psychiatry*.

[47] Tomoda A, Suzuki H, Rabi K, Sheu Y, Polcari A, Teicher MH (2009). Reduced prefrontal cortical gray matter volume in young adults exposed to harsh corporal punishment. *NeuroImage*.

[48] Benekareddy M, Goodfellow NM, Lambe EK, Vaidya VA (2010). Enhanced function of prefrontal serotonin 5-HT(2) receptors in a rat model of psychiatric vulnerability. *The Journal of Neuroscience*.

[49] Stevenson CW, Halliday DM, Marsden CA, Mason R (2008). Early life programming of hemispheric lateralization and synchronization in the adult medial prefrontal cortex. *Neuroscience*.

[50] Monroy E, Hernández-Torres E, Flores G (2010). Maternal separation disrupts dendritic morphology of neurons in prefrontal cortex, hippocampus, and nucleus accumbens in male rat offspring. *Journal of Chemical Neuroanatomy*.

[51] Muhammad A, Kolb B (2011). Maternal separation altered behavior and neuronal spine density without influencing amphetamine sensitization. *Behavioural Brain Research*.

[52] Pascual R, Zamora-León SP (2007). Effects of neonatal maternal deprivation and postweaning environmental complexity on dendritic morphology of prefrontal pyramidal neurons in the rat. *Acta Neurobiologiae Experimentalis*.

[53] Brenhouse HC, Andersen SL (2011). Nonsteroidal anti-inflammatory treatment prevents delayed effects of early life stress in rats. *Biological Psychiatry*.

[54] Chocyk A, Dudys D, Przyborowska A, Maćkowiak M, Wȩdzony K (2010). Impact of maternal separation on neural cell adhesion molecules expression in dopaminergic brain regions of juvenile, adolescent and adult rats. *Pharmacological Reports*.

[55] Arnold SE (1997). The medial temporal lobe in schizophrenia. *Journal of Neuropsychiatry and Clinical Neurosciences*.

[56] Szyf M (2009). The early life environment and the epigenome. *Biochimica et Biophysica Acta—General Subjects*.

[57] Massart R, Suderman M, Provencal N (2014). Hydroxymethylation and DNA methylation profiles in the prefrontal cortex of the non-human primate rhesus macaque and the impact of maternal deprivation on hydroxymethylation. *Neuroscience C*.

